# Microbiota and Metabolome Associated with Immunoglobulin A Nephropathy (IgAN)

**DOI:** 10.1371/journal.pone.0099006

**Published:** 2014-06-12

**Authors:** Maria De Angelis, Eustacchio Montemurno, Maria Piccolo, Lucia Vannini, Gabriella Lauriero, Valentina Maranzano, Giorgia Gozzi, Diana Serrazanetti, Giuseppe Dalfino, Marco Gobbetti, Loreto Gesualdo

**Affiliations:** 1 Department of Soil, Plant and Food Sciences, University of Bari Aldo Moro, Bari, Italy; 2 Department of Emergency and Organ Transplantation, Nephrology Unit - University of Bari Aldo Moro, Bari, Italy; 3 Department of Agricultural and Food Sciences, University of Bologna, Bologna, Italy; 4 Inter-departmental Centre for Industrial Agri-Food Research, University of Cesena, Cesena, Italy; Institut national de la santé et de la recherche médicale (INSERM), France

## Abstract

This study aimed at investigating the fecal microbiota, and the fecal and urinary metabolome of non progressor (NP) and progressor (P) patients with immunoglobulin A nephropathy (IgAN). Three groups of volunteers were included in the study: (i) sixteen IgAN NP patients; (ii) sixteen IgAN P patients; and (iii) sixteen healthy control (HC) subjects, without known diseases. Selective media were used to determine the main cultivable bacterial groups. Bacterial tag-encoded FLX-titanium amplicon pyrosequencing of the 16S rDNA and 16S rRNA was carried out to determine total and metabolically active bacteria, respectively. Biochrom 30 series amino acid analyzer and gas-chromatography mass spectrometry/solid-phase microextraction (GC-MS/SPME) analyses were mainly carried out for metabolomic analyses. As estimated by rarefaction, Chao and Shannon diversity index, the lowest microbial diversity was found in P patients. Firmicutes increased in the fecal samples of NP and, especially, P patients due to the higher percentages of some genera/species of Ruminococcaceae, Lachnospiraceae, Eubacteriaceae and Streptococcaeae. With a few exceptions, species of *Clostridium, Enterococcus* and *Lactobacillus* genera were found at the highest levels in HC. Bacteroidaceae, Porphyromonadaceae, Prevotellaceae and Rikenellaceae families differed among NP, P and HC subjects. Sutterellaceae and Enterobacteriaceae species were almost the highest in the fecal samples of NP and/or P patients. Compared to HC subjects, *Bifidobacterium* species decreased in the fecal samples of NP and P. As shown by multivariate statistical analyses, the levels of metabolites (free amino acids and organic volatile compounds) from fecal and urinary samples markedly differentiated NP and, especially, P patients.

## Introduction

Immunoglobulin A nephropathy (IgAN), also defined as mesangial IgA glomerulonephritis, is characterized by dominant mesangial IgA deposition. IgAN was first described by Berger in 1968 [1], and it is the most common primary glomerulonephritis worldwide [2], [3]. The prevalence of IgA nephropathy observed in renal biopsy registries is estimated to be 5% in the US, 10–20% in Europe and Australia, and 30–40% in Asia. Although it is a rather benign disorder in many patients, long-term studies showed that 25% to 50% of IgAN patients develop the end-stage kidney disease (ESKD) due to the progressive formation of IgA immune complexes (IgA-ICs) [4], [5], [6], [7], [8]. Although IgA-IC formation is thought to be universal in IgAN, clinical features, biopsy findings, and disease progression are highly variable. Patients in whom IgAN runs a benign course, the non progressors (NP), cannot currently be identified from progressors (P) at diagnosis [9]. Recently, IgAN patients were classified as non progressor (NP) when they fulfilled both the following criteria: (i) decrease, after at least 6 months, in daily proteinuria 50% of the value recorded at the start of therapy with Angiotensin-converting enzyme inhibitors (ACEi) treatment; and (ii) stable glomerular filtration rate throughout ACEi treatment [10], [11]. The IgAN pathogenesis is still unknown, and, even though under investigation, no specific treatments to stop the progression toward ESKD are currently available [12]. Hyper-production of poorly galactosylated IgA1 is thought to play a role in the primary IgAN [13]. The knowledge of downstream effector mechanisms, which are triggered by mesangial IgA1 deposition, is rather poor [14]. Genome-wide association studies (GWAS) identified multiple susceptibility loci IgAN, which implies independent defects of adaptive immunity (MHC region of the chromosome 6p21), innate immunity (8p23 DEFA, 17p23 TNFSF13 and 22q12 HORMAD2 loci) and alternative complement pathway (1q32 CFH/CFHR locus) [15]. Recently, it was shown the induction of glomerulonephritis by IgA rheumatoid factors [16]. Besides, the balance of various factors at the mucosal microenvironment level plays an important role in regulating the synthesis of IgA [17]. A presumptive role of the intestinal microbiota in educating the immune system and IgAN disease development was shown in B cell activation factor of the TNF family overexpressing trans-genic (BAFF-Tg) mice [14].

Overall, human microbiota contains 10^14^ bacterial cells, a number that is 10 times higher than the number of human body cells [18]. The human intestine is particularly dense of microbes, 10^12^ bacteria per g (dry weight), especially at colon level. The intestinal ecosystem is in part responsible for maintaining human health. It is involved in the protection against pathogens, education of the immune system and modulation of gastrointestinal development. Intestinal microbes play a pivotal role or contribute to many diseases. Alterations of the composition of the intestinal microbiota are associated with inflammatory bowel diseases (IBD), irritable bowel syndrome and allergic diseases [18], [19], [20], [21]. Differences on the composition of the intestinal microbiota are also linked to Type 1 and Type 2 diabetes [22], celiac disease (CD) [23], [24], autism [25], liver disease [26] and minimal hepatic encephalopathy (MHE) [27]. Under healthy conditions, the human microbiota provides trophic and protective functions. Nevertheless, uremia per se and therapeutic treatments of ESKD (i.e., phosphate binders, diet restriction) may lead to modifications of the composition and metabolic activities of the intestinal microbiota [28]. Recently, it was hypotized that the manipulation of the microbiota can be a strategy for the gastro-intestinal diseases [29], [30], [31]. Previously, it was shown that patients with ESKD had a marked increase of *Brachybacterium* and *Catenibacterium* genera, and Enterobacteriaceae, Halomonadaceae, Moraxellaceae, Nesterenkonia, Polyangiaceae, Pseudomonadaceae, and Thiothrix families within the intestinal microbiota [28], [32]. Despite of the considerable investigation of the biochemical abnormalities of IgA1, the composition and metabolic activity of human intestinal microbiota in IgAN patients were poorly considered. Signaling induced by TNF family members BAFF was implicated in the IgAN pathogenesis of BAFF-Tg mice [14]. Besides, BAFF-Tg mice developed commensal bacteria-IgA associated nephropathy. This evidence might support the role of the intestinal microbiota in the gut-kidney axis.

This study aimed to comparing IgAN patients (NP and P) and healthy controls (HC) based on fecal microbiota, and urinary and fecal metabolome. Total and active fecal microbiota was analyzed through an integrate approach of culture-dependent and -independent methods, and metabolomic analyses. Bacterial tag-encoded FLX-titanium amplicon pyrosequencing (bTEFAP) and gas-chromatography mass spectrometry/solid-phase microextraction (GC-MS/SPME) analyses were mainly carried out for genomic and metabolomic analyses.

## Materials and Methods

### Study design

The study was carried out in accordance with the Helsinki Declaration (IV Adaptation) and European Guidelines for Good Clinical Practice. The protocol of the study was approved by the Institutional Review Board of Azienda Ospedaliero-Universitaria Consorziale Policlinico of Bari, Italy. Written consents were obtained from all subjects. Volunteers were enrolled at the out-patient setting of the “Division of Nephrology, Dialysis and Transplant, Policlinico Hospital” of Bari, Italy. Three groups of caucasian volunteers were included in the study: (i) sixteen IgAN NP patients (subjects numbered: 1 – 16 NP); (ii) sixteen IgAN P patients (subjects numbered: 1 – 16 P); and (iii) sixteen healthy control (HC) subjects, without known diseases (subjects numbered: 1 – 16 HC). HC group included healthy subjects matched to cases on age and gender. Exclusion criteria included the presence of type 1 or type 2 diabetes mellitus, neurological or gastro-intestinal diseases, acute myocardial infarction or stroke in the previous 6 months, severe uncontrolled hypertension (diastolic blood pressure ≥120 mmHg and/or systolic blood pressure ≥220 mmHg), evidence or suspicion of renovascular disease, severe liver diseases, malignancies, active peptic-ulcer disease, secondary IgAN or relapse in renal allograft, renal allograft; pregnancy, others immunological or autoimmune disorders, alcohol abuse, psychiatric disease and inability to assess the follow-up. Subjects included in the study were not treated with antibiotics and/or functional foods (probiotics and/or prebiotics) for at least three months before sampling. All volunteers confirmed that there were no remarkable changes in meals and medication for at least 1 month. We evaluated serum creatinine, urine protein, glomerular filtration rate (MDRD GFR) and Body Mass Index at baseline and after six months of follow up (Table 1). In addition, renal biopsy specimens of all IgAN patients were classified according to Oxford Classification which identify four pathological features (mesangial hypercellularity M, endocapillary hypercellularity E, segmental glomerulosclerosis S, and tubular atrophy/interstitial fibrosis T, resulting in a MEST score). After six months, as described by Rocchetti et al. [11], patients were classified as NP and P according to their proteinuria and renal function. In detail, IgAN NP patients showed a 50% decrease in daily proteinuria and a stable renal function (eGFR) when compared with baseline. For HC, dipstick urine analysis (Combur-Test, Boehringer Mannheim GmbH, Mannheim, Germany) was carried out (Table 1).

**Table 1:** Basic characteristics of studied healthy controls (HC) and Immunoglobulin A nephropathy (IgAN) patients.

			Healthy controls (HC)						Non progressor (NP) group									Progressor (P) group				
									Baseline				End of Follow-up					Baseline			End of Follow-up	
Age (years)			43±8						41±10									45±6				
Male (%)			60						69									63				
Serum creatinine (mg/dL)			0.88±0.24^b^						1.07±0.22^b^				1.09±0.22^b^					2.39±1.18^a^			2.50±1.36^a^	
Proteinuria (g/day)			0.05±0.01^e^						0.47±0.3^c^				0.23±0.02^d^					0.71±0.81^b^			1.43±0.86^a^	
MDRD GFR (mL/min/1.73 m^2^)			96±7^a^						76±15^b^				77±14^b^					30±18^c^			37±19^c^	
Body Mass Index (kg/m^2^)			25±4						25±4				25±5					26±4			26±3	
**Full urine analysis of HC**																						
**Density**	**pH**			**Leukocytes**		**Blood/Haemoglobine**					**Nitrite**			**Ketones**			**Bilirubin**			**Urobilinogen**		**Protein**
1.016+/−0.013	6.5±0.9			negative		negative					negative			negative			negative			normal		negative
**Frequency of pathologic features (percentages) in 32-biopsies scored according to Oxford Classification (MEST)**																						
	**Mesangial hypercellularity (M)**				**Endocapillary proliferation (E)**					**Segmental glomerulosclerosis (S)**					**Tubular atrophy/interstitial fibrosis (T)**							
	M0 (%)		M1 (%)		E0 (%)			E1 (%)		S0 (%)		S1 (%)			T0 (%)				T1 (%)		T2 (%)	
NP	73^b^		27^c^		93^a^			7^e^		40^a^		60^e^			73^b^				20^d^		7^e^	
P	31^d^		69^b^		69^b^			31^d^		8^f^		92^a^			23^e^				46^c^		31^d^	
**Therapy**																						
		**HC**					**NP**									**P**						
ACE inhibitors (%)		0^b^					100^a^									100^a^						

a–e Values within a row with different superscript letters are significantly different (*P*<0.05).

### Collection of fecal and urine samples

Each volunteer provided three fecal and urinary samples for a time span of three weeks. Each volunteer had fasted overnight, and fecal sample was collected in the morning pre-prandial. Urine samples were collected after the second mittus into sterile collection cups. After collection, in sterile plastic box, feces were immediately mixed with RNA later (Sigma-Aldrich, St. Louis, MO, USA) (5 g, 1∶2 w/v) or with Amies Transport medium (Oxoid LTD, Basingstoke, Hampshire, England) (15 g, 1∶1 w/w), under anaerobic conditions (AnaeroGen, Oxoid LTD). Fecal samples suspended in RNA later were stored at −80°C for further DNA and RNA analyses. Samples diluted with Amies Transport medium were immediately subjected to culture-dependent (plate counts) and metabolome analyses. Three urine aliquots per each subject were immediately frozen and stored at −80°C until use.

### Enumeration of cultivable bacteria

Diluted fecal samples (20 g) were mixed with 80 ml sterilized physiological solution and homogenized. Counts of viable bacterial cell were carried out as described by Francavilla et al. [20]. The following selective media were used: Plate count agar (total facultative aerobes and anaerobes); MRS agar (lactobacilli and enterococci); *Bifidobacterium* agar modified (bifidobacteria) (Becton Dickinson France SA, Le Pont de Claix, France); M17 (lactococci and streptococci); Mannitol salt agar (stafilococci); Wilkins-Chalgren anaerobe agar (total anaerobes); Wilkins-Chalgren anaerobe agar plus GN selective supplements and sheep blood defibrinated (*Bacteroides*, *Porphyromonas* and *Prevotella*); Reinforced Clostridial Medium supplemented with 8 mg/L novobiocin, 8 mg/L colistin (*Clostridium*); MacConkey agar No2 (Enterobacteria); Rogosa agar plus 1.32 mL/L of glacial acetic acid (lactobacilli); GSP agar (Fluka) plus penicillin-G (60 g/L) (*Pseudomonas*, *Aeromonas*); and Slanetz and Bartley (enterococci). Except for *Bifidobacterium* agar modified and GSP agar, all media were purchased by Oxoid Ltd (Hampshire, England).

### DNA extraction from fecal samples

After homogenization with RNA later, fecal samples were mixed 1∶1 with distilled water in sterile plastic pestle. The homogenate was subjected to mechanical disruption with the FastPrep instrument (BIO 101) and total DNA was extracted with the FastDNA Spin Kit for Soil (MP Biomedicals, Illkrich, France), according to the manufacturer's instructions. An aliquot of about 300 µL of each fecal sample was diluted in 1 mL of PBS-EDTA (phosphate buffer 0.01 M, pH 7.2, 0.01 M EDTA). After centrifugation (14,000×*g* at 4°C for 5 min), the pellet was washed two times to decrease the content of PCR inhibitors. The resulting pellet was re-suspended into 300 µL of PBS-EDTA and used for DNA extraction with the FastPrep. The product was consisting of 50–100 µL of application-ready DNA. Quality and concentration of DNA extracts were determined using 1% agarose-0.5X TBE gels, which were stained with Gel Red 10,000X (Biotium, Inc.) and analyzed through spectrophotometric measurements at 260, 280 and 230 nm, using the NanoDrop ND-1000 Spectrophotometer (ThermoFisher Scientific Inc., MI, Italy).

### RNA extraction from fecal samples

An aliquot of 200 mg of fecal sample diluted in RNA later was used for RNA extraction with the Stool total RNA purification kit (Norgen, Thorold, TO). Total RNA was treated with RNase-free DNase I (Roche, Almere, Netherlands; 10 U of DNase per 20 µg of RNA) for 20 min at room temperature. Quality and concentration of RNA extracts were determined using 1% agarose-0.5X TBE gels and spectrophotometric measurements at 260, 280 and 230 nm through the NanoDrop ND-1000 Spectrophotometer. Total RNA extracted (2.5 µg) was retrotranscribed to cDNA using random examers and the Tetro cDNA synthesis kit from Bioline (Bioline, Freiburg, Germany), according to the manufacturer's instructions [33].

### Bacterial tag-encoded FLX amplicon pyrosequencing (bTEFAP) and data analyses

For each subject, the three DNA or cDNA samples, which corresponded to as any samplings, were pooled and used for bTEFAP analysis. bTEFAP was performed by Research and Testing Laboratories (Lubbock, TX), according to standard laboratory procedures and using the 454 FLX Sequencer (454 Life Sciences, Branford, CT). Samples were amplified for bTEFAP using a forward and reverse fusion primer. The forward primer was constructed with (5′–3′) the Roche A linker (CCATCTCATCCCTGCGTGTCTCCGACTCAG), an 8–10 bp barcode, and the forward 28F: GAGTTTGATCNTGGCTCAG primer [34]. The reverse fusion primer was constructed with (5′–3′) a biotin molecule, the Roche B linker (CCTATCCCCTGTGTGCCTTGGCAGTCTCAG), and reverse 519R GTNTTACNGCGGCKGCTG. Primers 28F and 519R are based upon the V1–V3 region (*Escherichia coli* position 27–519) of the 16S rRNA gene [34]. Amplifications were performed in 25 µl reactions with Qiagen HotStar Taq master mix (Qiagen Inc, Valencia, California), 1 µl of each 5 µM primer, and 1 µl of template. Reactions were performed on ABI Veriti thermocyclers (Applied Biosytems, Carlsbad, California) under the following thermal profile: 95°C for 5 min, then 35 cycles of 94°C for 30 sec, 54°C for 40 sec, 72°C for 1 min, followed by one cycle of 72°C for 10 min and 4°C hold. Amplification products were visualized with eGels (Life Technologies, Grand Island, NY). Products were then pooled equimolar and each pool was cleaned with Diffinity RapidTip (Diffinity Genomics, West Henrietta, NY), and size selected using Agencourt AMPure XP (BeckmanCoulter, Indianapolis, Indiana) following Roche 454 protocols (454 Life Sciences, Branford, Connecticut). Size selected pools were then quantified and 150 ng of DNA were hybridized to Dynabeads M-270 (Life Technologies) to create single stranded DNA following Roche 454 protocols (454 Life Sciences). Raw sequence data were screened, trimmed and filtered with default settings, using the QIIME pipeline version 1.4.0 (http://qiime.sourceforge.net). Chimeras were excluded by using the B2C2 (http://www.researchandtesting.com/B2C2.html) [35]. Sequences less than 250-bp were removed. Sample sets yielded 320,012 and 192,374 bacterial 16S rDNA and 16S rRNA gene sequences, respectively. After quality control, pyrosequencing analysis yielded an average of 8,355 reads sequences (average length 394-bp) per sample. Sequences that pass the quality control screening were condensed into a single FASTA formatted sequence and quality file such that each read has a one line descriptor followed by a single line of sequence/quality data. The descriptor line in both files has been altered to contain the samples name followed by the original descriptor line, separated with a unique delimiter (::). FASTA sequences for each sample, without chimeras, were evaluated using BLASTn against a database derived from GenBank (http://ncbi.nlm.nih.gov) [36].

### Taxonomic identification

The sequences were first clustered into Operational Taxonomic Unit (OTU) clusters with 97% identity (3% divergence), using USEARCH [37]. For each cluster the seed sequence was placed in a FASTA formatted file. To determine the identities of bacteria, sequences were first queried using a distributed BLASTn. NET algorithm [36] against a database of high-quality 16S bacterial sequences that derived from NCBI. Database sequences were characterized as high quality based on criteria, which were originally described by Ribosomal Database Project (RDP, v10.28) [38]. Using a NET and C# analysis pipeline, the resulting BLASTn outputs were compiled and validated using taxonomic distance methods, and data reduction analysis was performed as described previously [39]. Based on the above BLASTn derived sequence identity percentage, the sequences were resolved at the appropriate taxonomic levels as follows: ≥97% (<3% divergence), species level (OTUs); 95 – 97%, genus; 90 – 95%, family; 85 – 90%, order; 80 – 85%, class; and 77 – 80%, phyla. Any match below this identity levels was discarded. The percentage of each bacterial identification (ID) was individually analyzed for each fecal sample, providing relative abundance information on relative numbers of reads within a given sample. Divergences of 3 and 5% were indicative of sequences, which differed at species and genus levels, respectively. The student's t-test results seen in Table 2 indicate significantly lower diversity levels in NP and P when compared to HC. Alpha diversity (rarefaction, Good's coverage, Chao1 richness and Shannon diversity indices) and beta diversity measures were calculated and plotted using QIIME [34], [40], [41]. Final datasets at species and other relevant taxonomy levels were compiled into separate worksheets for compositional analysis among the three groups of subjects [42]. Differences in microbial communities between NP, P and HC groups were also investigated using the phylogeny-based unweighted Unifrac distance metric [34].

**Table 2:** Pyrosequencing data summary.

	HC group	NP group	P group
*16S rDNA*			
OTUs (species)	284±12.0^a^	232±11.3^b^	206±10.4^c^
Chao1	809±42.1^a^	738±31.8^b^	689±35.6^c^
Shannon index	5.13±0.16^a^	4.87±0.04^b^	4.52±0.11^b^
Good's coverage	98.36±1.02	98.45±1.87	97.16±1.93
*16S rRNA*			
OTUs (species)	256±13.08^a^	233±14.37^b^	214±17.20^c^
Chao1	782±40.27^a^	717±38.01^b^	643±26.84^c^
Shannon index	5.02±0.03^a^	4.61±0.05^b^	4.38±0.07^c^
Good's coverage	97.45±2.09	97.28±1.54	98.03±1.70

HC, healthy controls;NP, Immunoglobulin A nephropathy (IgAN) non-progressor; P, IgAN progressor; 16S rDNA, total bacteria; 16S rRNA, metabolically active bacteria.

a–c Values within a row with different superscript letters are significantly different (*P*<0.05).

### Fecal concentration of free amino acids

Total and individual free amino acids (FAA) of fecal samples were analyzed through the Biochrom 30 series amino acid analyzer (Biochrom Ltd., Cambridge Science Park, England) with a sodium cation-exchange column (20 by 0.46 cm [inner diameter]). A mixture of amino acids at known concentrations (Sigma Chemical Co., Milan, Italy) was added with cysteic acid, methionine sulfoxide, methionine sulfone, tryptophan, ornithine, glutamic acid, and γ-amino-butyric acid (GABA) and used as standard. Proteins and peptides in the samples were precipitated by addition of 5% (v/v) cold solid sulfosalicylic acid, holding the samples at 4°C for 1 h, and centrifuged them at 15,000×*g* for 15 min. The supernatant was filtered through a 0.22- µm-pore-size filter and when necessary diluted, with sodium citrate (0.2 M, pH 2.2) loading buffer. Amino acids were post-column derivatized with ninhydrin reagent and detected by absorbance at 440 (proline and hydroxyproline) or 570 (all the other amino acids) nm.

### Gas-chromatography mass spectrometry-solid-phase microextraction (GC-MS/SPME) analysis of fecal and urinary volatile compounds

After preconditioning, according to the manufacturer's instructions, a polydimethylsiloxane/Divinylbenzene fiber (PDMS/DVB) (65 µm) and a manual solid phase micro-extraction (SPME) holder (Supelco Inc., Bellefonte, PA) were used. Before headspace sampling, the fiber was exposed to GC inlet for 5 min for thermal desorption at 250°C. Three grams of fecal sample were placed into 10 mL glass vials and added of 10 µL of 4-methyl-2-pentanol (final concentration of 33 mg/L), as the internal standard. Samples were then equilibrated for 10 min at 40°C. SPME fiber was exposed to each sample for 40 min. Both equilibration and absorption phases were carried out with stirring. The fiber was then inserted into the injection port of the gas chromatograph for 10 min of sample desorption. GC-MS analyses were carried out with an Agilent 7890A gas chromatograph (Agilent Technologies, Palo Alto, CA) coupled to an Agilent 5975C mass selective detector operating in an electron impact mode (ionization voltage, 70 eV). A Supelcowax 10 capillary column (length, 60 m; inside diameter, 0.32 mm; Supelco, Bellefonte, PA) was used. The temperature program was 50°C for 1 min, followed by an increase, at a rate of 4.5°C/min, to 65°C, an increase, at a rate of 10°C/min, to 230°C, and then 230°C for 25 min. The injector, interface and ion source temperatures were 250, 250, and 230°C, respectively. The mass-to-charge ratio interval was 30 to 350 Da at a rate of 2.9 scans per s. Injection was carried out in splitless mode, and helium (flow rate, 1 mL/min) was used as the carrier gas. Molecule identification was based on comparison of their retention times with those of pure compounds (Sigma-Aldrich, Milan, Italy). Identities were confirmed by searching mass spectra in the available databases (NIST, version 2005; Wiley, version 1996). All the GC-MS raw files were converted to netCDF format via Chemstation (Agilent Technologies) and subsequently processed with the XCMS toolbox (http://metlin.scripps.edu/download/). XCMS software allows automatic and simultaneous retention time alignment, matched filtration, peak detection and peak matching. The resulting table containing information such as peak index (retention time-m/z pair) and normalized peak area was exported into R (www.r-project.org) for subsequent statistical or multivariate analyses [43]. Quantitative data for the compounds identified were obtained by the interpolation of the relative areas versus the internal standard area [44]. GC-MS/SPME data were organized into matrix and analyzed by Canonical discriminant Analysis of Principal coordinates [24].

### Statistical analysis

bTEFAP data (Unifrac distance metric and taxonomic abundance) were analyzed by Principal Component Analysis (PCA) to assess the bacterial composition of samples [42], [45], using the statistical software Statistica for Windows (Statistica 6.0 per Windows 1998, (StatSoft, Vigonza, Italia). Variables (features) are the relative bacterial composition in the sample at a particular taxonomic level [42]. In addition, the Permut-MatrixEN software was used to identify clusters at the level of the NP, P and HC groups and taxa [46]. Culture dependent and metabolomics data were obtained at least in triplicates. The analysis of variance (ANOVA) was carried out on transformed data followed by separation of means with Tukey's HSD, using the statistical software Statistica for Windows (Statistica 6.0 per Windows 1998, (StatSoft)). Letters indicate significant different groups (*P*<0.05) by Tukey's test. Canonical discriminant Analysis of Principal coordinates (CAP) analysis was also carried out for GC-MS/SPME data [44]. The hypothesis of not significant differences in the multivariate location within groups was tested using the trace statistic based on 9999 permutations [44].

## Results

### Enumeration of fecal cultivable bacteria

**Figure 1:**
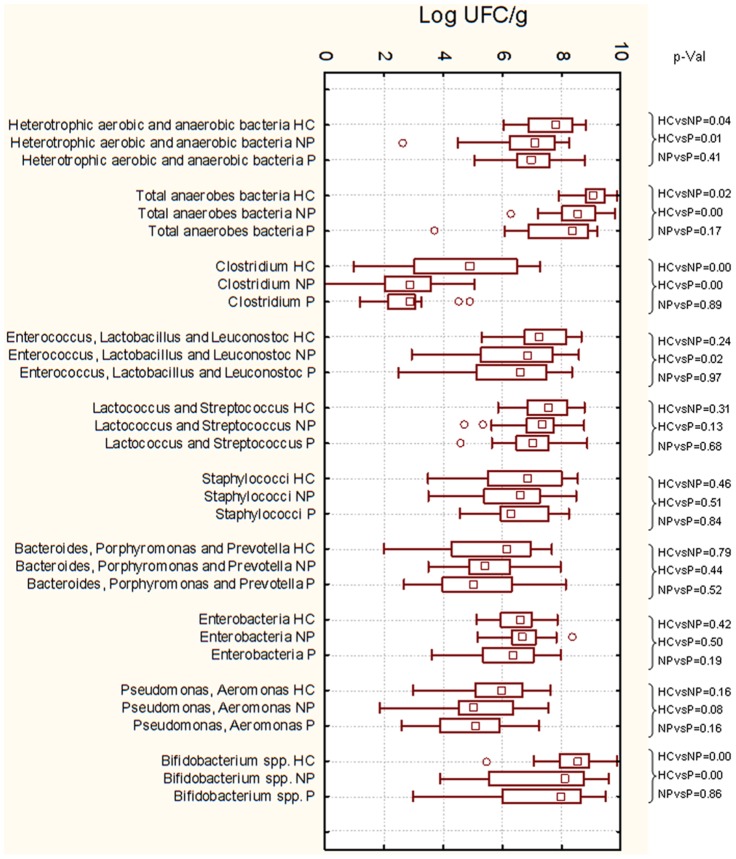
Fecal cultivable bacteria of the main microbial groups. Cultivable cells (log cfu/g) found in the fecal samples of immunoglobulin A nephropathy (IgAN) non progressor (NP) and progressor (P) patients, and healthy controls (HC). Data are the means of three independent experiments (*n* = 3). The top and bottom of the box represent the 75th and 25th percentile of the data, respectively. The top and bottom of the error bars represent the 5th and 95th percentile of the data, respectively. ○, Outliers data. Group student's t-test p-values were also shown.

Compared to HC, the number of heterotrophic aerobic and anaerobic bacteria significantly (*P*<0.05) decreased in the fecal samples of NP and P patients (Figure 1). Total presumptive *Clostridium* were the highest in HC. P patients showed median values of presumptive *Enterococcus*, *Lactobacillus* and *Leuconostoc* lower than those found for HC and NP. No statistical differences (*P*>0.05) were found between NP, P and HC for presumptive *Lactococcus, Streptococcus, Staphylococcus, Bacteroides* and Enterobacteriaceae. Compared to HC, significantly (*P*<0.05) lower counts of presumptive *Bifidobacterium* were found in the fecal samples of NP and, especially, P patients.

### Pyrosequencing analysis of the fecal community of bacteria

**Figure 2:**
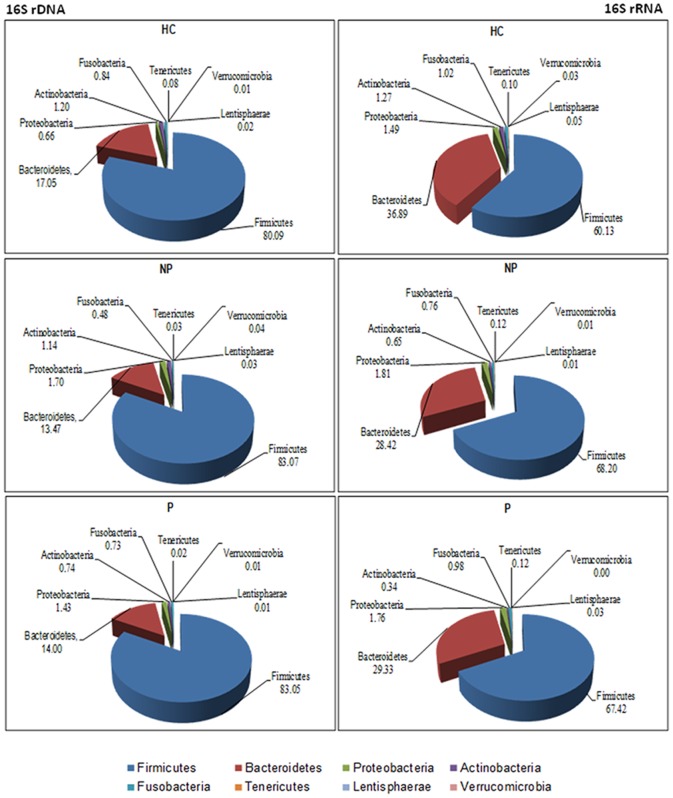
Total and active bacteria found in feces of subjects. Relative abundance (%) of total (16S rDNA) and metabolically active (16S rRNA) bacteria, which were found at the phylum level in the fecal samples of immunoglobulin A nephropathy (IgAN) non progressor (NP) and progressor (P) patients, and healthy controls (HC).

**Figure 3:**
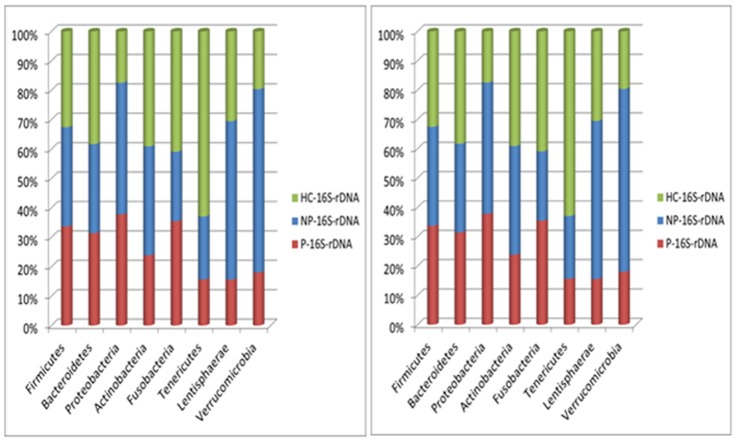
Comparison of total and active bacterial phyla found in feces of subjects. Bacterial phyla distribution (%) found in fecal samples of immunoglobulin A nephropathy (IgAN) non progressor (NP) and progressor (P) patients, and healthy controls (HC). The X-axis represents the proportion of phyla from total (16S rDNA) and active (16S rRNA) bacteria.

Total and metabolically active bacteria from fecal samples of NP and P patients and HC subjects were analyzed by pyrosequencing of 16S rDNA and rRNA genes, respectively. The bacterial community was analyzed by rarefaction curves, species level (OTUs) richness estimator (Chao1) and diversity index (Shannon) (Table 3). The average number of OTUs indicated differences between fecal samples of NP and P compared to HC. Eight bacterial phyla were identified in NP, P and HC subjects (Figure 2 and 3). Firmicutes, Bacteroidetes, Proteobacteria and Actinobacteria represented more than 98% of all 16S rDNA and 16S rRNA gene sequences (Figure 2 and Figure S1). Overall, different values were found between total (16S rDNA analysis) (Figure 2 and S1A) and metabolically active phyla (16S rRNA analysis) (Figure 2 and S1B). No significant (*P*>0.05) differences were found for total Firmicutes between NP, P and HC (83.07, 83.05 and 80.09%, respectively) (Figure 2 and S1A). Compared to HC (60.13%), metabolically active Firmicutes increased in NP (68.20%) and P (67.42%) patients (Figure 2 and S1B). An opposite trend was found for Bacteroidetes, which showed the highest value in HC in both 16S rDNA and 16S rRNA analyses (Figure 2 and and S1). Total and metabolically active Proteobacteria and Actinobacteria were, respectively, the lowest and the highest in HC.

**Table 3:** Immunoglobulin A nephropathy (IgAN) associated microbiome.

Phylum/Family	Specie	Avg (%) HC	Avg (%) NP	Avg (%) P
Firmicutes/Ruminococcaceae	*Ruminococcus flavefaciens*	0.16^a^	0.18^a^	0.06^b^
	*Ruminococcus gnavus*	0.48^a^	0.29^a^	0.15^b^
	*Ruminococcus obeum*	0.07^b^	0.20^a^	0.11^a^
	*Anaerofilum* sp.	0.13^a^	0.06^b^	0.11^a^
	*Oscillospira* sp.	3.79^a^	2.56^a,b^	1.75^b^
	*Sporobacter termitidis*	0.07^b^	0.17^a^	0.17^a^
	*Subdoligranulum* sp.	0.30^b^	0.86^a^	0.51^a,b^
	*Anaerotruncus* sp.	0.02^b^	0.07^a^	0.06^a^
	*Ethanoligenens harbinense*	0.03^b^	0.21^a^	0.03^b^
	*Hydrogenoanaerobacterium saccharovorans*	0.04^b^	0.14^a^	0.04^b^
	*Papillibacter cinnamivorans*	0.03^b^	0.08^a,b^	0.13^a^
Firmicutes/Clostridiaceae	*Clostridium aminophilum*	0.93^a,b^	0.72^b^	1.36^a^
	*Clostridium bolteae*	0.51^b^	1.25^a^	0.78^a,b^
	*Clostridium clostridioforme*	0.59^a^	0.22^b^	0.24^b^
	*Clostridium herbivorans*	0.12^b^	0.25^a^	0.24^a^
	*Clostridium methylpentosum*	0.49^a^	0.43^a^	0.13^b^
	*Clostridium nexile*	0.48^a,b^	0.68^a^	0.23^b^
	*Clostridium symbiosum*	0.37^a^	0.32^a^	0.22^a,b^
	*Clostridium xylanolyticum*	0.05^b^	0.12^a^	0.12^a^
Firmicutes/Lachnospiraceae	*Roseburia faecis*	0.84^c^	1.86^b^	3.31^a^
	*Roseburia intestinalis*	0.59^b^	4.12^a^	0.49^b^
	*Roseburia inulinivorans*	0.15^b^	0.22^a,b^	0.38^a^
	*Dorea* sp.	0.90^a,b^	1.24^a^	1.13^a^
	*Coprococcus eutactus*	0.88^a^	0.44^b^	0.24^c^
	*Coprococcus* sp.	0.32^a^	0.13^b^	0.33^a^
	*Lachnospira pectinoschiza*	1.01^a^	0.84^a,b^	0.54^b^
	*Butyrivibrio crossotus*	0.30^c^	2.34^a^	0.78^b^
Firmicutes/Eubacteriaceae	*Eubacterium desmolans*	0.05^b^	0.09^a^	0.10^a^
	*Eubacterium eligens*	1.04^a^	0.68^a,b^	0.45^b^
	*Eubacterium oxidoreducens*	0.22^a^	0.04^c^	0.07^b^
	*Eubacterium ramulus*	0.14^a,b^	0.20^a^	0.10^b^
	*Eubacterium siraeum*	0.99^a^	0.28^b^	0.22^b^
	*Eubacterium* sp.	5.81^b^	5.96^b^	8.57^a^
Firmicutes/Lactobacillaceae	*Lactobacillus rogosae*	0.20^b^	0.43^a^	0.19^b^
	*Lactobacillus* sp.	0.23^a^	0.00^b^	0.00^b^
Firmicutes/Streptococcaceae	*Streptococcus salivarius*	0.03^b^	0.01^b^	0.10^a^
	*Streptococcus* sp.	0.05^b^	0.05^b^	0.15^a^
Firmicutes/Erysipelotrichaceae	*Catenibacterium* sp.	0.37^a^	0.16^b^	0.22^a,b^
	*Eubacterium biforme*	0.14^a^	0.01^b^	0.00^b^
	*Turicibacter* sp.	0.01^c^	0.11^a^	0.04^b^
Firmicutes/Peptococcaceae	*Desulfotomaculum* sp.	0.30^a,b^	0.42^a^	0.20^b^
Firmicutes/Veillonellaceae	*Dialister* sp.	0.38^a^	0.18^b^	0.26^a,b^
	*Phascolarctobacterium* sp.	0.24^a^	0.14^a,b^	0.10^b^
Bacteroidetes/Bacteroidaceae	*Bacteroides coprocola*	1.67^b,c^	4.07^a^	2.21^b^
	*Bacteroides faecis*	0.17^b^	0.28^a,b^	0.41^a^
	*Bacteroides finegoldii*	0.23^a^	0.03^c^	0.10^b^
	*Bacteroides fragilis*	0.10^a^	0.13^a^	0.04^b^
	*Bacteroides intestinalis*	0.15^a^	0.00^b^	0.15^a^
	*Bacteroides ovatus*	0.25^a^	0.11^b^	0.18^a,b^
	*Bacteroides plebeius*	1.18^a^	0.27^b^	1.79^a^
	*Bacteroides salyersiae*	0.05^b^	0.03^b^	0.23^a^
	*Bacteroides* sp.	17.54^a^	13.59^a^	14.32^a^
	*Bacteroides thetaiotaomicron*	0.10^a^	0.07^a,b^	0.05^b^
	*Bacteroides uniformis*	2.24^a^	0.52^b^	0.75^b^
	*Bacteroides vulgatus*	3.29^a^	3.28^a^	1.40^b^
	*Pseudoflavonifractor capillosus*	0.07^b^	0.08^b^	0.16^a^
Bacteroidetes/Porphyromonadaceae	*Barnesiella intestinihominis*	0.29^a,b^	0.16^b^	0.45^a^
	*Butyricimonas virosa*	0.15^a^	0.03^c^	0.07^b^
	*Parabacteroides distasonis*	0.21^b,c^	0.84^a^	0.37^b^
Bacteroidetes/Prevotellaceae	*Prevotella copri*	1.80^a^	0.96^a,b^	1.13^a^
	*Prevotella* sp.	2.35^a^	0.61^b^	2.06^a^
Bacteroidetes/Rikenellaceae	*Alistipes putredinis*	1.67^a^	0.89^a,b^	1.12^a^
	*Alistipes* sp.	0.27^a^	0.16^a^	0.17^a^
Proteobacteria/Rhodospirillaceae	*Rhodospirillum rubrum*	0.08^a^	0.01^b^	0.12^a^
	*Rhodospirillum* sp.	0.17^a^	0.01^c^	0.05^b^
	*Thalassospira* sp.	0.03^b^	0.02^b^	0.06^a^
Proteobacteria/Alcaligenaceae	*Sutterella* sp.	0.23^b^	0.54^a^	0.57^a^
Proteobacteria/Sutterellaceae	*Parasutterella excrementihominis*	0.03^b^	0.17^a^	0.17^a^
Proteobacteria/Enterobacteriaceae	*Escherichia coli*	0.02^b^	0.07^a,b^	0.12^a^
	*Escherichia* sp.	0.02^c^	0.04^b^	0.15^a^
	*Proteus* sp.	0.29^b^	0.86^a^	0.30^b^
	*Shigella* sp.	0.01^b^	0.01^b^	0.15^a^
	*Enterobacter* sp.	0.01^b^	0.08^a^	0.11^a^
Actinobacteria/Bifidobacteriaceae	*Bifidobacterium* sp.	2.59^a^	1.21^b^	0.11^c^
Actinobacteria/Coriobacteriaceae	*Collinsella aerofaciens*	0.14^b^	0.48^a^	0.15^b^

Relative proportions of predominant metabolically active bacteria (16S rRNA) showing significant (*P*<0.05) differences between fecal samples of healthy controls (HC), non progressor (NP) and IgAN progressor (P).

a–c Values within a row with different superscript letters are significantly different (*P*<0.05).

### Distinctive core microbiome associated with IgAN

Compared to HC and NP, total (16S rDNA analysis) Streptococcaceae (phylum Firmicutes), markedly increased (*P*<0.05) in P patients and reached the 11.54% of the total 16S rDNA reads (Figure S1A). Eubacteriaceae family was higher in P compared to HC (Figure S1A–B). Lactobacillaceae was the lowest (*P*<0.05) in P patients. Within Bacteroidetes phylum, Bacteroidaceae and Prevotellaceae families were the lowest (*P*<0.05) in P and NP patients, respectively. Within the Proteobacteria, Alcaligenaceae and Enterobacteriaceae families increased (*P*<0.05) in P patients. Compared to HC, Bifidobacteriaceae (phylum Actinobacteria), markedly decreased (*P*<0.05) in NP and, especially, in P patients. At 16S rDNA level, Coriobacteriaceae increased in NP and P compared to HC. Differences in the relative abundance between total and metabolically active bacterial families were highlighted by permutation analyses (Figure S2). Overall, the highest similarity was found between HC and NP for total bacterial families, and between NP and P for metabolically active families.

**Figure 4:**
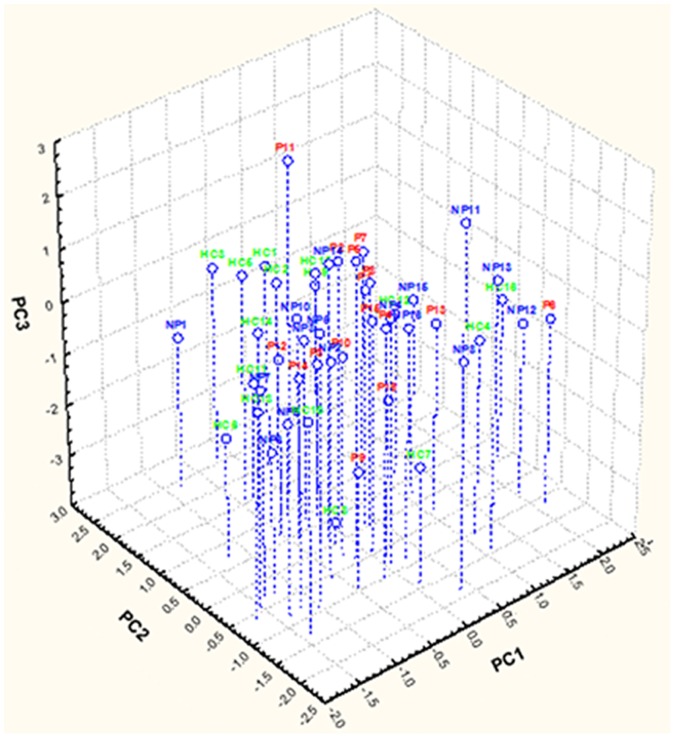
Principal component analysis (PCA) of total bacterial genera found in feces of subjects. Score plot of the three principal components (PC) after principal component analysis (PCA) of total bacterial genera (16S rDNA), which were found in the fecal samples of immunoglobulin A nephropathy (IgAN) non progressor (NP) and progressor (P) patients, and healthy controls (HC). 1–16, number of fecal samples for each group of subjects.

**Figure 5:**
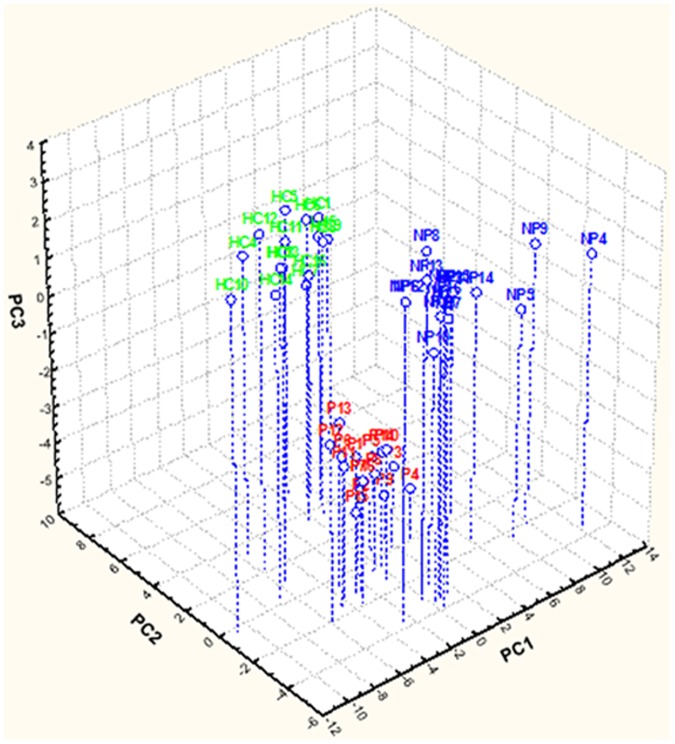
Principal component analysis (PCA) of active bacterial genera found in feces of subjects. Score plot of the three principal components (PC) after principal component analysis (PCA) of metabolically active bacterial genera (16S rRNA), which were found in the fecal samples of immunoglobulin A nephropathy (IgAN) non progressor (NP) and progressor (P) patients, and healthy controls (HC). 1–16, number of fecal samples for each group of subjects.

Using genus composition data, 223 and 192 genera were found by 16S rDNA and rRNA analyses, respectively. Unifrac distance metric and taxonomic abundance at genus level were further analyzed by PCA. As shown in the 3-D plot (Figure 4), no systematic differences were found among 16S rDNA sequences, which were associated to the three groups. On the contrary, fecal samples of NP, P and HC were clustered in different zone of the 3-D plot when the 16S rRNA sequences were analyzed (Figure 5). Based on this finding and considering that only metabolically active bacteria give a general picture of the microbial activities at the intestinal level, further analyses were carried out only on 16S rRNA data. The differences (*P*<0.05) in the relative abundance of OTU associated with the NP, P or HC subjects are described in Table 3. Within Firmicutes, all the 9 families showed at least one species that statistically (*P*<0.05) differed between NP, P or HC. Similar trends were found for the other three main phyla.

### Free amino acids (FAA) profiling of feces

**Figure 6:**
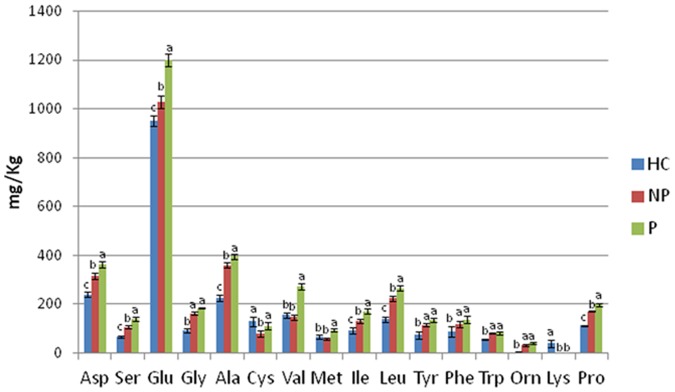
Fecal levels of free amino acids (FAA) in subjects. Concentration (mg/kg) of individual free amino acids (FAA) found in the fecal samples of immunoglobulin A nephropathy (IgAN) non progressor (NP) and progressor (P) patients, and healthy controls (HC). Data are the means of three independent experiments and standard deviations, performed in duplicate (*n* = 6).

The highest (*P*<0.05) level of total FAA was found in the fecal samples of P patients (3781 vs. 3124 and 2520 mg/kg for P vs. NP and HC, respectively). As shown in Figure 6, Glu, Ala, Asp, Val, Leu and Pro were the dominant FAA. Fecal samples of HC contained the highest concentration of Cys and Lys (128 and 39 mg/kg, respectively). Besides, GABA, which is synthesized through decarboxylation of Glu, was found at the highest concentration in the fecal samples of NP patients (8.52 vs. 5.72 and 5.69 mg/kg of feces for NP vs. P and HC, respectively). α-Ketoglutaric acid was present at the lowest level in the fecal samples of P patients (179 mg/kg). The concentration of ammonia (NH_3_) ranged from 98 (P) to 80 (HC) g/kg of fecal samples (data not shown).

### Volatile organic compounds (VOC) profiling of feces and urines

**Figure 7:**
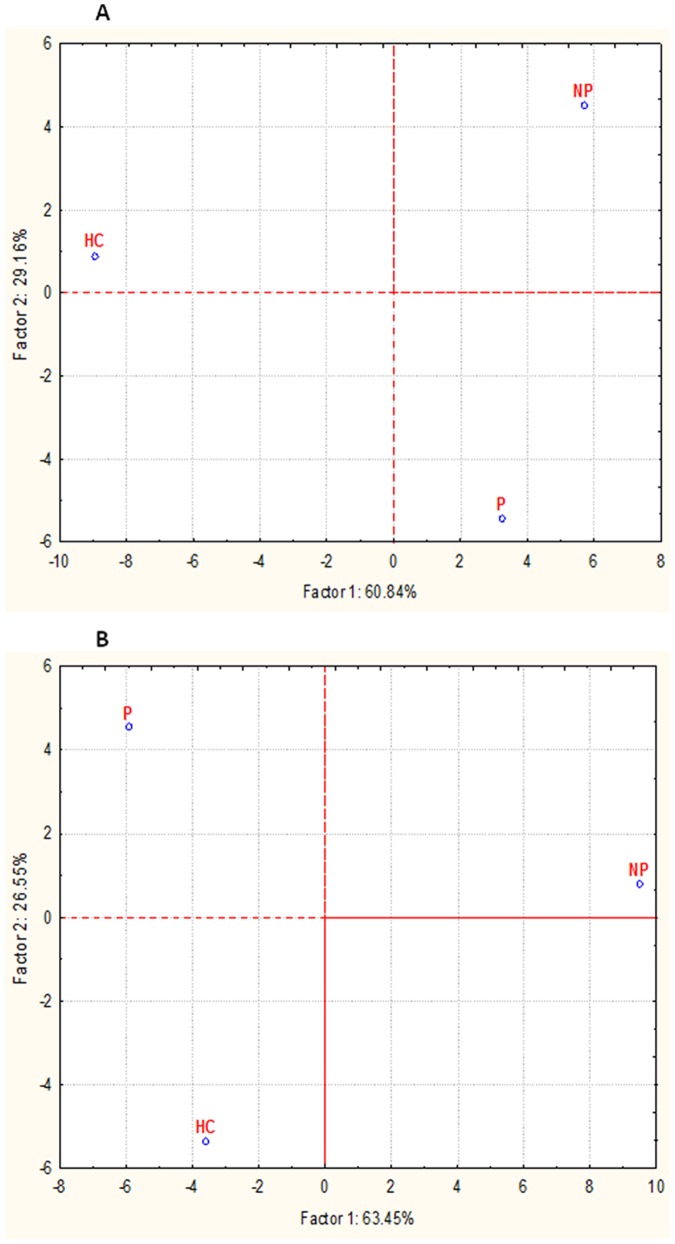
Principal component analysis (PCA) of volatile organic metabolites found in feces of subjects. Score plots of the two principal components (PC) after principal component analysis (PCA) of volatile organic metabolites of the fecal (A) and urine (B) samples of immunoglobulin A nephropathy (IgAN) non progressor (NP) and progressor (P) patients, and healthy controls (HC).

VOC were identified from fecal (74 compounds) and urine (73 compounds) samples, and grouped according to chemical classes: alcohols (10 and 15 compounds were identified from feces and urine, respectively), aldehydes (7 and 10), esters (22 and 4), aromatic heterocyclic (5 and 4), hydrocarbons (14 and 16), ketones (8 and 16), short and medium chain fatty acids (SCFA) (5 and 4), sulfur compounds (2 and 2) and terpens (1 and 2) (Table S1). Overall, the content of the various metabolites largely varied within the same group. Compared to HC, some compounds (ethyl alcohol, 2,6-octadien-1-ol 3,7 dimethyl- (Z), 1-octanol, 4-methyl-phenol and phenol 4-(1,1,3,3-tetramethylbutyl) were found at significantly (*P*<0.05) higher levels in the feces and/or urines of NP and, especially, P patients. Within aldehydes, tridecanal showed the highest level in the fecal samples of HC (Table S1). Nonanal, benzaldehyde and heptanal were found at the highest levels in the urine samples of NP patients. Except for pentanoic acid methyl ester, heptanoic acid 1, 1-dimethylethyl ester, hexyl n-valerate, heptanoic acid 1-methylethyl ester, benzoic acid hexadecyl ester, and phthalic acid methyl neopentyl ester, the levels of esters were significantly (*P*<0.05) higher in the fecal samples of NP and P than in those from HC (Table S1). Within urine samples, ethyl acetate and phthalic acid methyl neopentyl ester were at the highest levels in P and HC, respectively. Aromatic heterocyclic were variously distributed within groups. Except for benzene 1,4-bis(1, 1-dimethylethyl), hydrocarbons showed higher levels in the fecal samples of NP patients with respect to HC (Table S1). Compared to HC, hydrocarbons were also significantly (*P*<0.05) higher in the urine samples of P patients. The levels of ketons were significantly (*P*<0.05) lower in the fecal samples of HC compared to those found in NP and, especially, P patients (Table S1). Compared to HC, SCFA (acetic, propanoic, butanoic and pentanoic acids) were significantly (*P*<0.05) higher in the fecal samples of NP and P patients (Table S1). Carbon disulfide showed the highest concentration in the fecal samples of HC, while sulfur compounds were variously distributed within in the urine samples of the three groups (Table S1). GC-MS/SPME data were also analyzed by multivariate statistical approaches (Canonical discriminant Analysis of Principal coordinates, CAP and Principal Component Analysis, PCA). Both the analyses showed a clear separation between the three groups of subjects (See Figures 7A and 7B and data not shown).

## Discussion

Various extra-intestinal non-communicable diseases are associated with the microbiota through intestinal immunity [18], [47], [48]. Nevertheless, to the best of our knowledge no data are available regarding the composition of the intestinal microbiota of immunoglobulin A nephropathy (IgAN) patients [49]. Recently, a marked damage of the colonic epithelial barrier structure and an alteration of the colonic microbiota were shown in humans and animals with advanced chronic kidney disease (CKD) [28], [32], [50], [51]. The present study showed, for the first time, that some traits of the gut microbiota significantly varied between IgAN non-progressors (NP) and progressors (P) patients and healthy controls (HC). Moreover, it was showed that urinary and fecal metabolomic profiles, differed between study groups.

As determined by culture-dependent methods, cell densities of the total and anaerobe bacteria decreased in the fecal samples of NP and, especially, P patients. The lowest levels of presumptive *Clostridium*, *Enterococcus*, *Lactobacillus*, *Leuconostoc* and *Bifidobacterium* were found in the fecal samples of NP and, especially in P.

The fecal microbiota was further studied by culture-independent methods. According to previous results on induced chronic renal failure (CRF) rats [28], the fecal microbial diversity decreased in NP and, especially, in P patients. Overall, a similar trend was also found in IBD and allergic patients [52], [53]. This study showed that the composition of the main bacterial phyla (Firmicutes, Bacteroidetes, Proteobacteria and Actinobacteria) significantly differed in the fecal microbiota of NP, P and HC subjects.

Primer selection can influence the profile of a community generated by sequencing but the level of this bias on deep-sequencing methodologies is not well elucidated. While several investigations suggest that V1-V3 regions overestimate species richness, others support the use of these regions for deep sequencing [34], [42], [45]. As shown by multivariate statistical analyses, the main differences between NP, P and HC were concerning metabolically active bacteria (16S rRNA data). Regarding total bacteria, the main difference was found for the Streptoccocaceae family. The highest percentage of 16S rDNA reads belonging to Streptococcaeae was found in P patients. Using 16S rRNA, the number of Streptococcaeae reads decreased. This might be related to the survival during the gastrointestinal transit. A link between tonsillar infection caused by some salivary bacteria, including *Streptococcus* sp., and IgAN was hypothesized [54]. Besides, streptococcal proteins (e.g., M and GroEL) were present in tissues or they were recognized by sera of IgAN patients [55], [56].

Based on metabolically active bacteria (16S rRNA data), Firmicutes increased in the fecal samples of NP and, especially, P patients. The ratio between Firmicutes/Bacteroidetes may dramatically change by antibiotics, dietary nutrients and/or diseases [18]. The increase of Firmicutes was mainly related to the highest percentages of some genera/species of Lachnospiraceae (*Roseburia faecis*, *Dorea* sp., *Butyrivibrio crossotus*) and Eubacteriaceae (*Eubacterium* sp.). The Lachnospiraceae family also increased in induced CRF rats [28]. According to the results from culture-dependent methods, *Enterococcus* and *Lactobacillus* were found at the highest levels in HC. Overall, *Lactobacillus* is the main probiotic genus within lactic acid bacteria. The composition of Bacteroidaceae, Porphyromonadaceae, Prevotellaceae and Rikenellaceae families differed among NP, P and HC subjects. Nevertheless, *Bacteroides fragilis* group, which synthesizes lipopolysaccharide (LPS) as an important bacterial virulence factor [42], did not increase in the fecal samples of NP and/or P patients. According to a previous report on CRF or ESRD patients [28], [57], the level of Sutterellaceae and Enterobacteriaceae species (LPS producing bacteria) were almost the highest in the fecal samples of NP and/or P patients. In addition, DNA from *Enterobacter*, *Escherichia* and *Proteus* species was found in the blood of ESRD patients [58]. The content of *Bifidobacterium* species decreased in the fecal samples of NP and, especially, P patients. *Bifidobacterium* species were demonstrated to have species- and strain-specific influence on immunity [59]). Various species of *Bifidobacterium* synthesize exo-polysaccharides, which act as fermentable substrates for human intestinal bacteria [60].

As shown by metagenomic studies, the intestinal microbiota is directly involved on the metabolism of proteins, FAA and carbohydrates [18]. Overall, metabolites produced by intestinal microbes play a direct role in health and disease [61], [62]). A very few studies considered the metabolome of IgAN patients and no reports are available for human fecal samples [49], [63]. Previously, it was shown that serum samples of IgAN patients had an altered level of some metabolites, including an increase of some FAA (e.g., Asp, Glu, Gly, Ala, Val, Met, Ile, Leu, Tyr, Phe and Pro) [49], [50]. Compared to HC, a marked increase of total FAA was found in the fecal samples of NP and, especially, P patients. It was hypothesized that the IgAN pathology causes a marked alteration of the Krebs cycle and the increased hydrolysis of proteins from cell necrosis, which affects the level of FAA in serum. The absorption of proteins seems to be decreased in CRF patients, thus increasing the amount of proteins that are available for microbial proteolysis [64]. An alteration of the fecal microbiota composition, which was related to an increased level of FAA, was described in colon carcinoma patients [65].

The highest level of alcohols was found in the fecal samples of P patients. 4-Methyl-phenol (p-cresol) seemed to be over-synthesized in IgAN patients. High level of p-cresol and p-cresyl sulfate, which derive from the microbial hydrolysis of tyrosine, were associated with ESRD and CKD [66]. Highest levels of alcohols were also found in CD children [23], [24]. A positive correlation was found between the synthesis of alcohols by intestinal bacteria and endotoxemia [67]. Besides, some long-chain aliphatic alcohols (C6–C20) may inhibit the growth of various bacteria [68]. Compared to HC, the lowest level of aldehydes was found in the fecal and urine samples of P patients. On the contrary, urine samples of NP patients showed the highest level of aldehydes. Based on these findings, different catabolic pathways for FAA may be suggested for the subjects of study. Furthermore, the level of esters and ketons strongly increased in the fecal samples of NP and P patients. No previous data are available for IgAN, CKD and ESRD patients. Esterification reactions at the colon level are considered like a microbial strategy to remove toxic compounds [69]. The conversion of fatty acids into ketons and glucose increased in the hepatic cells to supply energy for peripheral cells, which are unable to transport glucose in the absence of insulin [22]. This hypothesis was also supported through the increase of SCFA (e.g., acetic acid), which represent the end products of the lipid metabolism [49]. Compared to fecal samples of HC, NP and P patients showed significantly higher levels of total SCFA (acetic, propanoic, butanoic and pentanoic acids).

IgAN patients had an altered fecal microbiota and VOC profiling, which in part differed between NP and P. The fecal microbiota and the FAA and VOC profiling of NP were more similar to HC than those of P patients. Dietary implementation with prebiotics and/or probiotic could be a useful tool to restore some microbial gaps (e.g., lactobacilli and bifidobacteria). Since this is a transversal study, further investigations are necessary to highlight the exact relationships between some microbial groups and IgAN.

## Supporting Information

Figure S1 **Total and active bacteria found in feces of subjects.** Relative abundance (%) of total (16S rDNA) (A) and metabolically active (16S rRNA) (B) Firmicutes, Bacteroidetes, Proteobacteria and Actinobacteria and related families, which were found in the fecal samples of immunoglobulin A nephropathy (IgAN) non progressor (NP) and progressor (P) patients, and healthy controls (HC).
(TIF)

Figure S2 **Permutation analysis.** Permutation analysis of the total (16S rDNA) and metabolically active (16S rRNA) bacterial families composition found in fecal samples of immunoglobulin A nephropathy (IgAN) non progressor (NP) and progressor (P) patients, and healthy controls (HC).
(TIF)

Table S1 Concentration (ppm) of volatile organic compounds (VOC).
(DOCX)
