# Supplementary figures and images for: Microbiota and Metabolome Associated with Immunoglobulin A Nephropathy (IgAN)
Source: PLoS One. 2014 Jun 12;9(6):e99006. doi: 10.1371/journal.pone.0099006 (PMC4055632; doi:10.1371/journal.pone.0099006)

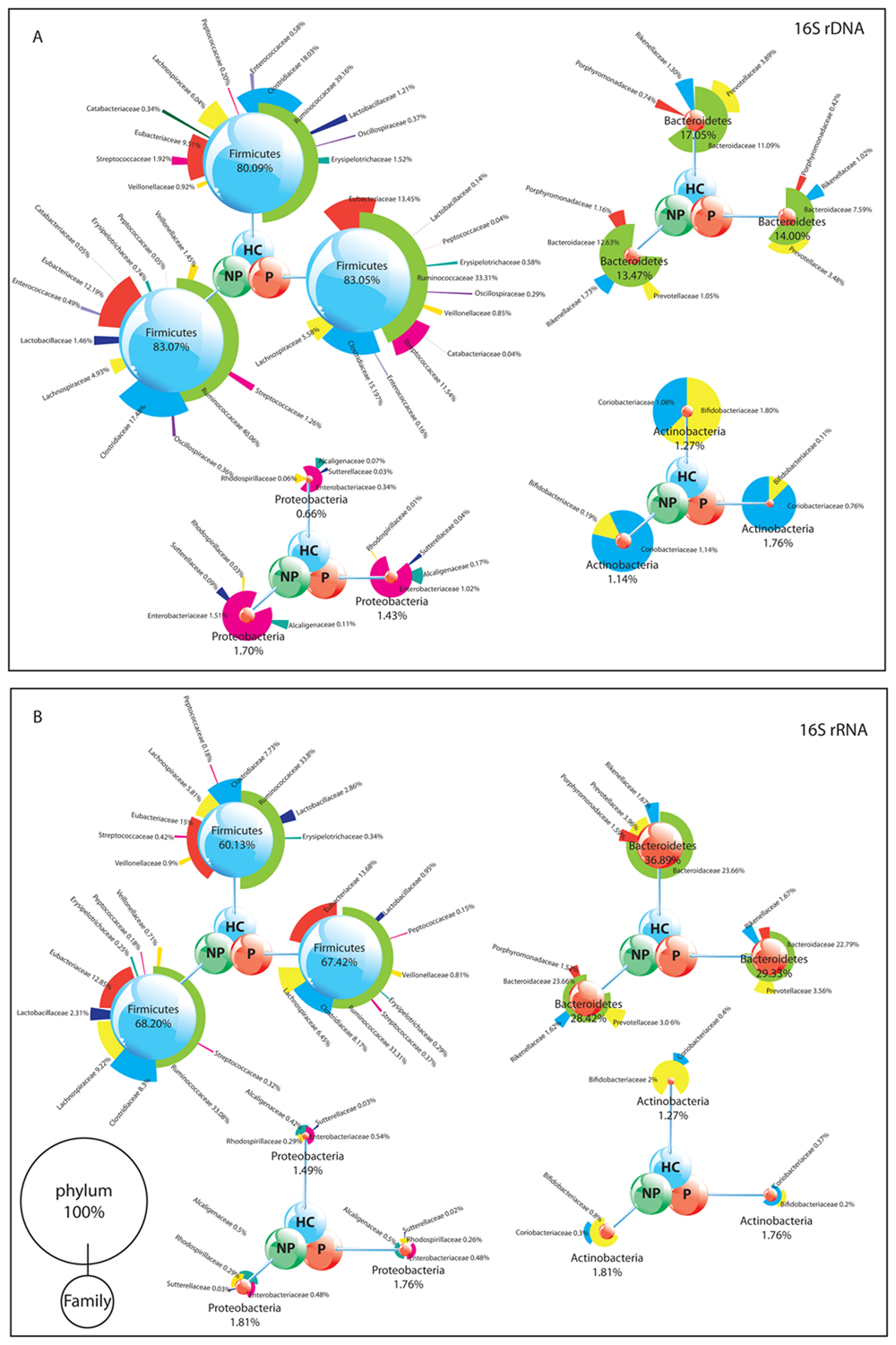

Supplement: Figure S1 — Total and active bacteria found in feces of subjects. Relative abundance (%) of total (16S rDNA) (A) and metabolically active (16S rRNA) (B) Firmicutes, Bacteroidetes, Proteobacteria and Actinobacteria and related families, which were found in the fecal samples of immunoglobulin A nephropathy (IgAN) non progressor (NP) and progressor (P) patients, and healthy controls (HC). (TIF) [file pone.0099006.s001.tif]

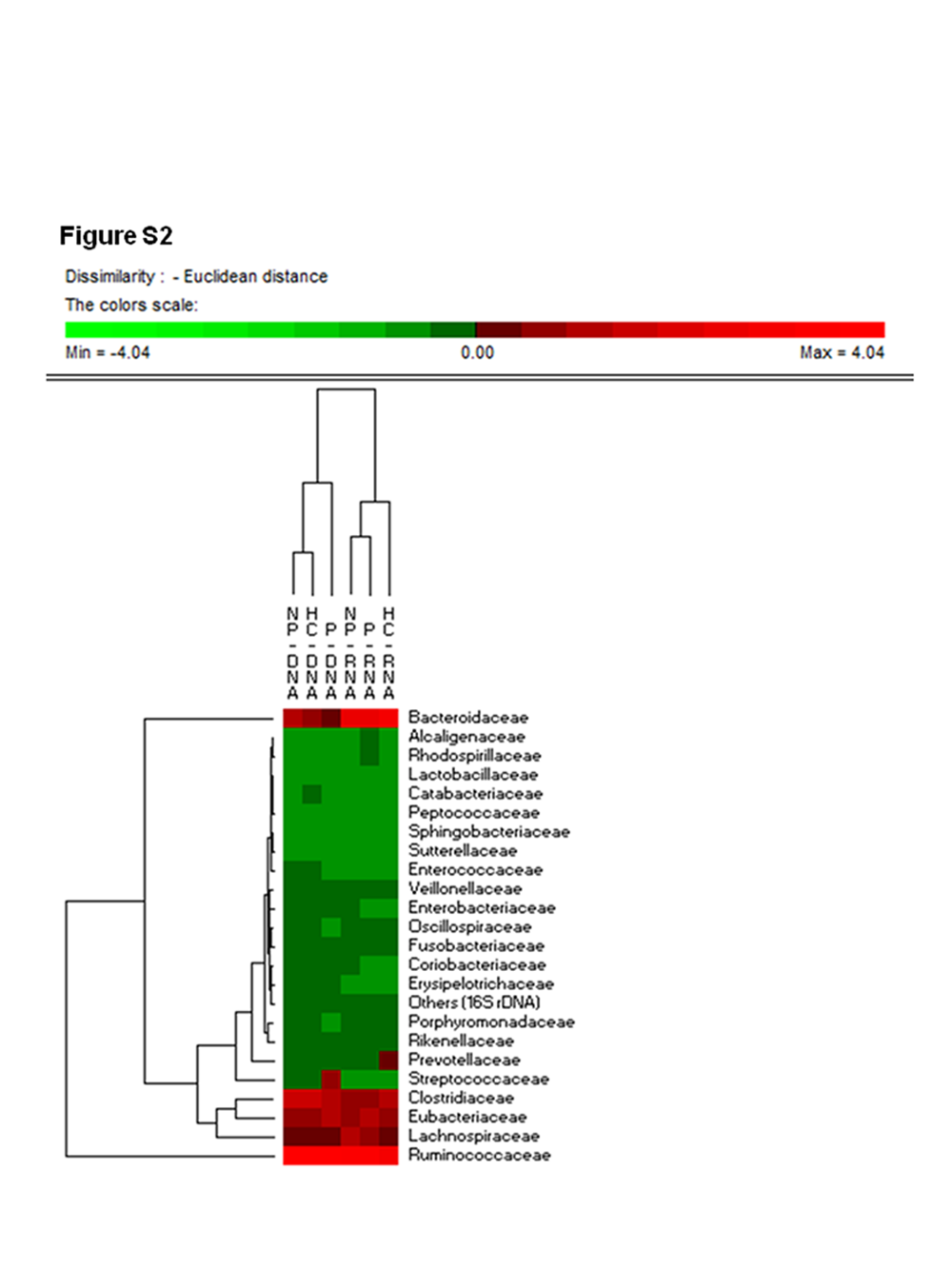

Supplement: Figure S2 — Permutation analysis. Permutation analysis of the total (16S rDNA) and metabolically active (16S rRNA) bacterial families composition found in fecal samples of immunoglobulin A nephropathy (IgAN) non progressor (NP) and progressor (P) patients, and healthy controls (HC). (TIF) [file pone.0099006.s002.tif]
