# Supplementary material for: Microbiota and Metabolome Associated with Immunoglobulin A Nephropathy (IgAN)
Source: PLoS One. 2014 Jun 12;9(6):e99006. doi: 10.1371/journal.pone.0099006 (PMC4055632; doi:10.1371/journal.pone.0099006)
Supplement: Table S1 — Concentration (ppm) of volatile organic compounds (VOC). (DOCX) [file pone.0099006.s003.docx]

| **Chemical class** | **HC** | | **NP** | | **P** | |
| --- | --- | --- | --- | --- | --- | --- |
|  | Feces | Urine | Feces | Urine | Feces | Urine |
| Alcohols |  |  |  |  |  |  |
| Ethyl alcohol | 0.40^d^ | 1.52^b^ | 1.03^b^ | 0.94^c^ | 2.15^a^ | 2.31^a^ |
| 1-Butanol | 0.05^c^ | 0.00^d^ | 0.19^a^ | 0.00^d^ | 0.09^b^ | 0.00^d^ |
| 2,6-Octadien-1-ol, 3,7 dimethyl- (Z) | 0.77^b^ | 0.00^c^ | 0.65^b^ | 0.00^c^ | 3.79^a^ | 0.00^c^ |
| 1-Pentanol | 0.03^a^ | 0.01^b^ | 0.04^a^ | 0.00^c^ | 0.03^a^ | 0.00^c^ |
| 1-Octanol | 0.02^b^ | 0.02^b^ | 0.02^b^ | 0.04^b^ | 0.14^a^ | 0.01^b^ |
| 1-Trydecin-4-ol | 0.09^a^ | 0.00^b^ | 0.06^a^ | 0.00^b^ | 0.07^a^ | 0.00^b^ |
| 1-[2-Methyl-3-(methylthio)allyl]cyclohex-2-enol | 0.60^a^ | 0.81^a^ | 0.36^b^ | 0.43^b^ | 0.60^a^ | 0.86^a^ |
| 4-Methyl-phenol | 0.93^c^ | 0.00^d^ | 1.36^a^ | 0.00^d^ | 1.03^b^ | 0.00^d^ |
| Phenol, p-tert-butyl- | 0.09^d^ | 0.70^a^ | 0.04^d^ | 0.33^c^ | 0.08^d^ | 0.58^b^ |
| Phenol, 4-(1,1,3,3-tetramethylbutyl)- | 0.57^c^ | 1.54^b^ | 0.18^c^ | 1.39^b^ | 0.39^c^ | 1.98^a^ |
| 1,4-Benzenediol, 2,5-bis(1,1-dimethylethyl) | 0.00^b^ | 0.55^a^ | 0.00^b^ | 0.39^a^ | 0.00^b^ | 0.68^a^ |
| 3-Heptanol | 0.00^c^ | 0.13^b^ | 0.00^c^ | 0.27^a^ | 0.00^c^ | 0.18^ab^ |
| 3-Methyl-phenol | 0.00^c^ | 0.01^b^ | 0.00^c^ | 0.02^a^ | 0.00^c^ | 0.02^a^ |
| 5-Methyl-3-hexanol | 0.00^b^ | 0.03^a^ | 0.00^b^ | 0.03^a^ | 0.00^b^ | 0.02^a^ |
| Diisobutyl 4,5-dimethylphthalate/5-methyl-2,4-diisopropylphenol | 0.00^b^ | 0.15^a^ | 0.00^b^ | 0.14^a^ | 0.00^b^ | 0.16^a^ |
| Phenol, 2-(1,1dimethyethyl)-4-(1,1,3,3-tetramethylbutyl)- | 0.00^c^ | 0.31^a^ | 0.00^c^ | 0.18^b^ | 0.00^c^ | 0.29^a^ |

**Table S1**. Concentration (ppm) of volatile organic compounds (VOC).

| **Chemical class** | **HC** | | **NP** | | **P** | |
| --- | --- | --- | --- | --- | --- | --- |
|  | Feces | Urine | Feces | Urine | Feces | Urine |
| Phenol, 2-methyl-4-(1,1,3,3-tetramethylbutyl)- | 0.00^c^ | 0.33^a^ | 0.00^c^ | 0.19^b^ | 0.00^c^ | 0.31^a^ |
| Phenol, 2,5-bis(1,1-dimethylethyl) | 0.00^c^ | 0.40^a^ | 0.00^c^ | 0.17^b^ | 0.00^c^ | 0.33^a^ |
| Phenol, m-tert-butyl | 0.00^b^ | 0.01^a^ | 0.00^b^ | 0.01^a^ | 0.00^b^ | 0.01^a^ |
| Total alcohols | 3.55^f^ | 6.52^c^ | 3.93^e^ | 4.53^d^ | 8.37^a^ | 7.74^b^ |
| Aldehydes |  |  |  |  |  |  |
| Octanal | 0.02^b^ | 0.21^a^ | 0.02^b^ | 0.25^a^ | 0.01^b^ | 0.18^a^ |
| Nonanal | 0.07^c^ | 0.83^b^ | 0.09^c^ | 1.83^a^ | 0.07^c^ | 0.81^b^ |
| Decanal | 0.01^a^ | 0.01^a^ | 0.02^a^ | 0.01^a^ | 0.01^a^ | 0.01^a^ |
| Benzaldehyde | 0.05^b^ | 0.06^b^ | 0.04^b^ | 0.13^a^ | 0.04^b^ | 0.05^b^ |
| Tridecanal | 1.04^a^ | 0.00^d^ | 0.30^b^ | 0.00^d^ | 0.17^c^ | 0.00^d^ |
| Pentadecanal | 0.01^a^ | 0.00^b^ | 0.01^a^ | 0.00^b^ | 0.01^a^ | 0.00^b^ |
| Hexadecanal | 0.01^a^ | 0.00^b^ | 0.01^a^ | 0.00^b^ | 0.01^a^ | 0.00^b^ |
| 2-Butyl-2-octenal | 0.00^b^ | 0.01^a^ | 0.00^b^ | 0.02^a^ | 0.00^b^ | 0.01^a^ |
| 2-Methyl-benzaldheyde | 0.00^c^ | 0.20^a^ | 0.00^c^ | 0.07^b^ | 0.00^c^ | 0.08^b^ |
| 2-Octenal | 0.00^b^ | 0.02^a^ | 0.00^b^ | 0.02^a^ | 0.00^b^ | 0.01^a^ |
| 2,4-Heptadienal, 2,4-dimethyl | 0.00^c^ | 0.23^a^ | 0.00^c^ | 0.13^b^ | 0.00^c^ | 0.25^a^ |
| Heptanal | 0.00^c^ | 0.42^b^ | 0.00^c^ | 0.49^a^ | 0.00^c^ | 0.37^b^ |
| Hexanal | 0.00^c^ | 0.62^a^ | 0.00^c^ | 0.62^a^ | 0.00^c^ | 0.41^b^ |
| Total aldehydes | 1.21^d^ | 2.61^b^ | 0.49^e^ | 3.57^a^ | 0.32^f^ | 2.18^c^ |

| **Chemical class** | **HC** | | **NP** | | **P** | |
| --- | --- | --- | --- | --- | --- | --- |
|  | Feces | Urine | Feces | Urine | Feces | Urine |
| Esters |  |  |  |  |  |  |
| Acetic acid, methyl ester | 0.14^b^ | 0.00^c^ | 0.36^a^ | 0.00^c^ | 0.27^a^ | 0.00^c^ |
| Ethyl acetate | 0.09^d^ | 0.15^c^ | 0.80^a^ | 0.05^e^ | 0.82^a^ | 0.25^b^ |
| Methyl propionate | 0.83^b^ | 0.00^d^ | 1.05^c^ | 0.00^d^ | 1.35^a^ | 0.00^d^ |
| Propanoic acid, ethyl ester | 0.18^b^ | 0.00^c^ | 1.60^a^ | 0.00^c^ | 1.80^a^ | 0.00^c^ |
| N-propyl acetate | 0.05^b^ | 0.00^c^ | 0.75^a^ | 0.00^c^ | 0.74^a^ | 0.00^c^ |
| Butanoic acid, methyl ester | 3.04^b^ | 0.00^c^ | 5.76^a^ | 0.00^c^ | 5.52^a^ | 0.00^c^ |
| Butanoic acid, ethyl ester | 0.39^b^ | 0.00^c^ | 3.67^a^ | 0.00^c^ | 3.87^a^ | 0.00^c^ |
| Propanoic acid, propyl ester | 0.01^b^ | 0.00^c^ | 0.11^a^ | 0.00^c^ | 0.12^a^ | 0.00^c^ |
| Acetic acid, butyl ester | 0.01^c^ | 0.00^d^ | 0.28^a^ | 0.00^d^ | 0.04^b^ | 0.00^d^ |
| Pentanoic acid, methyl ester | 0.53^a^ | 0.00^c^ | 0.31^a^ | 0.00^c^ | 0.37^a^ | 0.00^c^ |
| Butanoic acid, propyl ester | 0.28^bc^ | 0.00^c^ | 0.82^a^ | 0.00^c^ | 0.49^ab^ | 0.00^c^ |
| Pentanoic acid, ethyl ester | 0.01^b^ | 0.00^b^ | 0.08^a^ | 0.00^b^ | 0.12^a^ | 0.00^b^ |
| Heptanoic acid, 1,1-dimethylethyl ester | 0.06^a^ | 0.06^a^ | 0.09^a^ | 0.05^ab^ | 0.07^a^ | 0.08^a^ |
| Butanoic acid, hexyl ester | 0.00^b^ | 0.00^b^ | 0.04^a^ | 0.00^b^ | 0.02^a^ | 0.00^b^ |
| Butanoic acid, 3methylbutyl ester | 0.00^b^ | 0.00^b^ | 0.01^a^ | 0.00^b^ | 0.01^a^ | 0.00^b^ |
| Hexyl n-valerate | 0.01^a^ | 0.00^b^ | 0.02^a^ | 0.00^b^ | 0.01^a^ | 0.00^b^ |
| Heptanoic acid, 1-methylethyl ester | 0.05^a^ | 0.04^a^ | 0.05^a^ | 0.04^a^ | 0.04^a^ | 0.04^a^ |
| Cyclohexane carboxylic acid, ethyl ester | 0.00^c^ | 0.00^c^ | 0.06^a^ | 0.00^c^ | 0.01^b^ | 0.00^c^ |

| **Chemical class** | **HC** | | **NP** | | **P** | |
| --- | --- | --- | --- | --- | --- | --- |
|  | Feces | Urine | Feces | Urine | Feces | Urine |
| Cyclohexane carboxylic acid, pentyl ester | 0.00^c^ | 0.00^c^ | 0.13^a^ | 0.00^c^ | 0.02^b^ | 0.00^c^ |
| Cyclohexane carboxylic acid, heptyl ester | 0.01^b^ | 0.00^b^ | 0.07^a^ | 0.00^b^ | 0.01^b^ | 0.00^b^ |
| Benzoic acid, hexadecyl ester | 0.08^a^ | 0.00^b^ | 0.04^b^ | 0.00^b^ | 0.06^ab^ | 0.00^b^ |
| Phthalic acid, methyl neopentyl ester | 0.22^b^ | 0.71^a^ | 0.12^c^ | 0.62^a^ | 0.19^b^ | 0.64^a^ |
| Total esters | 5.99^b^ | 0.96^c^ | 16.22^a^ | 0.76^c^ | 15.95^a^ | 1.01^c^ |
| Aromatic heterocyclic |  |  |  |  |  |  |
| Furan | 0.30^a^ | 0.04^b^ | 0.23^a^ | 0.03^b^ | 0.19^a^ | 0.03^b^ |
| 2-Pentyl-furan | 0.44^b^ | 0.49^b^ | 1.75^a^ | 1.53^a^ | 0.42^b^ | 0.52^b^ |
| Furanone A | 2.82^a^ | 0.00^d^ | 1.46^c^ | 0.00^d^ | 2.34^b^ | 0.00^d^ |
| Indole | 0.10^b^ | 0.00^c^ | 0.10^b^ | 0.00^c^ | 0.16^a^ | 0.00^c^ |
| 3-Methyl-indole | 0.12^a^ | 0.00^d^ | 0.07^b^ | 0.00^d^ | 0.04^c^ | 0.00^d^ |
| 2-Methylfuran | 0.00^b^ | 0.05^a^ | 0.00^b^ | 0.04^a^ | 0.00^b^ | 0.04^a^ |
| Pyrrole | 0.00^d^ | 1.10^a^ | 0.00^d^ | 0.34^b^ | 0.00^d^ | 0.19^c^ |
| Total aromatic heterocyclic | 3.78^a^ | 1.68^c^ | 3.61^a^ | 1.94^c^ | 3.15^b^ | 0.78^d^ |
| Hydrocarbons |  |  |  |  |  |  |
| Octane | 0.07^b^ | 0.00^e^ | 0.26^a^ | 0.01^d^ | 0.10^b^ | 0.00^e^ |
| 2,4-Dimethyl-1-heptene | 0.27^b^ | 0.00^c^ | 0.91^a^ | 0.00^c^ | 0.86^a^ | 0.00^c^ |
| Benzene | 0.80^a^ | 0.08^b^ | 0.93^a^ | 0.06^b^ | 1.02^a^ | 0.05^b^ |

| **Chemical class** | **HC** | | **NP** | | **P** | |
| --- | --- | --- | --- | --- | --- | --- |
|  | Feces | Urine | Feces | Urine | Feces | Urine |
| Trichloromethane | 0.62^a^ | 0.26^b^ | 0.64^a^ | 0.63^a^ | 0.56^a^ | 0.68^a^ |
| Toluene | 0.25^b^ | 0.00^c^ | 0.35^a^ | 0.00^c^ | 0.42^a^ | 0.00^c^ |
| O-xylene | 0.07^a^ | 0.00^b^ | 0.08^a^ | 0.00^b^ | 0.10^a^ | 0.00^b^ |
| 3-Methyl-2-undecene (Z) | 0.21^b^ | 0.00^c^ | 0.41^a^ | 0.00^c^ | 0.23^b^ | 0.00^c^ |
| 2-Butanonene,4-hydroxy-3-methyl | 0.02^a^ | 0.00^b^ | 0.03^a^ | 0.00^b^ | 0.03^a^ | 0.00^b^ |
| Benzene, 1-methyl-2-(1-methylethyl)- | 0.63^c^ | 0.00^a^ | 2.05^a^ | 0.00^a^ | 1.27^b^ | 0.00^a^ |
| 1-Chloro-hexane | 0.00^b^ | 0.06^a^ | 0.00^b^ | 0.07^a^ | 0.00^b^ | 0.06^a^ |
| 1-Chloro-octane | 0.00^c^ | 0.14^b^ | 0.00^c^ | 0.24^a^ | 0.00^c^ | 0.13^b^ |
| 1-Methyl-2-(1-methylethyl)-benzene | 0.00^d^ | 0.03^c^ | 0.00^d^ | 0.12^a^ | 0.00^d^ | 0.06^b^ |
| 1,3-Hexadiene, 3-ethyl-2-methyl | 0.00^b^ | 0.01^a^ | 0.00^b^ | 0.01^a^ | 0.00^b^ | 0.01^a^ |
| 2-Pentene/2-methyl-1-butene | 0.00^b^ | 0.07^a^ | 0.00^b^ | 0.12^a^ | 0.00^b^ | 0.10^a^ |
| 2,3-(Methylbutyl)-thiophene | 0.00^b^ | 0.03^a^ | 0.00^b^ | 0.02^a^ | 0.00^b^ | 0.02^a^ |
| 5-Methyl-1,4-hexadiene | 0.00^b^ | 0.07^a^ | 0.00^b^ | 0.05^a^ | 0.00^b^ | 0.07^a^ |
| Hexadecane | 0.00^b^ | 0.02^b^ | 0.00^b^ | 0.06^a^ | 0.00^b^ | 0.01^b^ |
| Naphthalene, 1,2,3,4-tetrahydro- 1,1,6-trimethyl | 0.00^b^ | 0.02^a^ | 0.00^b^ | 0.02^a^ | 0.00^b^ | 0.02^a^ |
| O-diacetylbenzene | 0.00^b^ | 0.07^a^ | 0.00^b^ | 0.05^a^ | 0.00^b^ | 0.07^a^ |
| Styrene | 0.00^b^ | 0.01^a^ | 0.00^b^ | 0.01^a^ | 0.00^b^ | 0.01^a^ |

| **Chemical class** | **HC** | | **NP** | | **P** | |
| --- | --- | --- | --- | --- | --- | --- |
|  | Feces | Urine | Feces | Urine | Feces | Urine |
| Thiophene | 0.00^c^ | 0.12^b^ | 0.00^c^ | 0.05^b^ | 0.00^c^ | 1.21^a^ |
| 6-Methyl-1-heptene | 0.08^a^ | 0.00^c^ | 0.06^a^ | 0.00^c^ | 0.03^b^ | 0.00^c^ |
| Cyclohexane carboxylic acid | 0.01^a^ | 0.00^b^ | 0.02^a^ | 0.00^b^ | 0.00^b^ | 0.00^b^ |
| Benzene, 1,4-bis(1,1-dimethylethyl)- | 3.96^a^ | 0.35^c^ | 2.58^b^ | 0.20^d^ | 2.33^b^ | 0.50^c^ |
| 2-Pentyl-thiophene | 0.01^a^ | 0.00^b^ | 0.01^a^ | 0.00^b^ | 0.01^a^ | 0.00^b^ |
| Cyclohexane carboxylic acid (o simil) | 0.00^b^ | 0.00^b^ | 0.01^a^ | 0.00^b^ | 0.01^a^ | 0.00^b^ |
| Total hydrocarbons | 7.00^b^ | 1.34^e^ | 8.34^a^ | 1.72^d^ | 6.97^b^ | 3.00^c^ |
| Ketones |  |  |  |  |  |  |
| Acetone | 1.40^e^ | 3.39^c^ | 2.21^d^ | 5.43^b^ | 2.24^d^ | 7.35^a^ |
| 2-Butanone | 0.86^e^ | 2.43^c^ | 3.93^b^ | 1.26^d^ | 5.51^a^ | 4.00^b^ |
| Methyl isobutyl ketone | 0.05^a^ | 0.06^a^ | 0.07^a^ | 0.08^a^ | 0.06^a^ | 0.07^a^ |
| 4-Heptanone | 0.01^d^ | 4.88^b^ | 0.02^d^ | 7.51^a^ | 0.03^d^ | 3.22^c^ |
| 4-Methyl-3-penten-2-one | 0.63^b^ | 1.59^a^ | 0.68^b^ | 1.80^a^ | 0.77^b^ | 1.63^a^ |
| 2,6-Dimethyl-4-heptanone | 0.13^c^ | 0.77^b^ | 0.16^c^ | 1.50^a^ | 0.11^c^ | 1.61^a^ |
| 1-Phenyl-2-hexanone | 0.14^b^ | 0.00^c^ | 0.42^a^ | 0.00^c^ | 0.43^a^ | 0.00^c^ |
| N-octyl phenyl ketone | 0.04^a^ | 0.00^b^ | 0.04^a^ | 0.00^b^ | 0.04^a^ | 0.00^b^ |
| 2-Cyclohexen-1-one, 2-methyl- 5-(1-methylethenyl)- (S) | 0.00^c^ | 0.28^a^ | 0.00^c^ | 0.33^a^ | 0.00^c^ | 0.16^b^ |
| 2-Heptanone | 0.00^b^ | 0.09^a^ | 0.00^b^ | 0.12^a^ | 0.00^b^ | 0.07^a^ |

| **Chemical class** | **HC** | | **NP** | | **P** | |
| --- | --- | --- | --- | --- | --- | --- |
|  | Feces | Urine | Feces | Urine | Feces | Urine |
| 2-Methyl-3-decen-5-one | 0.00^c^ | 2.83^a^ | 0.00^c^ | 1.79^b^ | 0.00^c^ | 3.08^a^ |
| 2-Nonanone | 0.00^b^ | 0.02^a^ | 0.00^b^ | 0.01^a^ | 0.00^b^ | 0.02^a^ |
| 2-Pentanone | 0.00^c^ | 3.46^a^ | 0.00^c^ | 3.89^a^ | 0.00^c^ | 2.17^b^ |
| 2,5-Octanedione | 0.00^b^ | 0.02^a^ | 0.00^b^ | 0.02^a^ | 0.00^b^ | 0.01^a^ |
| 3-Butyl-3-octen-2-one | 0.00^c^ | 0.17^a^ | 0.00^c^ | 0.09^b^ | 0.00^c^ | 0.16^a^ |
| 3-Hexanone | 0.00^b^ | 0.01^a^ | 0.00^b^ | 0.01^a^ | 0.00^b^ | 0.01^a^ |
| 4-Methyl-2-hexanone | 0.00^a^ | 4.06^b^ | 0.00^a^ | 6.14^a^ | 0.00^a^ | 2.87^c^ |
| 4,6-Dimethyl-2-heptanone | 0.00^b^ | 0.02^a^ | 0.00^b^ | 0.02^a^ | 0.00^b^ | 0.02^a^ |
| Total ketones | 3.26^e^ | 24.08^b^ | 7.53^d^ | 30.00^a^ | 9.19^c^ | 26.45^b^ |
| Short chain fatty acids |  |  |  |  |  |  |
| Acetic acid | 0.09^b^ | 0.04^b^ | 0.46^a^ | 0.08^b^ | 0.61^a^ | 0.05^b^ |
| Propanoic acid | 0.06^b^ | 0.04^b^ | 0.26^a^ | 0.04^b^ | 0.37^a^ | 0.04^b^ |
| Butanoic acid | 0.30^b^ | 0.00^c^ | 1.89^a^ | 0.00^c^ | 1.74^a^ | 0.00^c^ |
| Pentanoic acid | 0.13^b^ | 0.00^c^ | 0.31^a^ | 0.00^c^ | 0.41^a^ | 0.00^c^ |
| Hexanoic acid | 0.14^a^ | 0.00^b^ | 0.13^a^ | 0.00^b^ | 0.11^a^ | 0.00^b^ |
| 3-Methyl-2-propionyl-benzoic acid | 0.00^b^ | 0.10^a^ | 0.00^b^ | 0.06^a^ | 0.00^b^ | 0.11^a^ |
| Nonanoic acid | 0.00^c^ | 0.03^b^ | 0.00^c^ | 0.07^a^ | 0.00^c^ | 0.03^b^ |
| Total short chain fatty acids | 0.72^b^ | 0.21^c^ | 3.05^a^ | 0.25^c^ | 3.24^a^ | 0.23^c^ |
| Sulfur compounds |  |  |  |  |  |  |
| Carbon disulfide | 77.50^a^ | 0.00^c^ | 64.76^b^ | 0.00^c^ | 67.07^b^ | 0.00^c^ |
| Disulfide, dimethyl | 0.41^b^ | 0.06^d^ | 1.00^a^ | 0.10^c^ | 1.04^a^ | 0.03^e^ |

| **Chemical class** | **HC** | | **NP** | | **P** | |
| --- | --- | --- | --- | --- | --- | --- |
|  | Feces | Urine | Feces | Urine | Feces | Urine |
| Dimethyl trisulfide | 0.00^c^ | 0.02^b^ | 0.00^c^ | 0.08^a^ | 0.00^c^ | 0.01^b^ |
| Total sulfur compounds | 77.91^a^ | 0.08^d^ | 65.76^b^ | 0.18^c^ | 68.11^b^ | 0.04^d^ |
| Terpenes |  |  |  |  |  |  |
| Menthol | 0.02^a^ | 0.01^a^ | 0.02^a^ | 0.01^a^ | 0.02^a^ | 0.01^a^ |
| (+)-(Z)-Longipinane | 0.00^b^ | 0.73^a^ | 0.00^b^ | 0.41^a^ | 0.00^b^ | 0.78^a^ |
| Total terpenes | 0.02^c^ | 0.74^a^ | 0.02^c^ | 0.42^b^ | 0.02^c^ | 0.79^a^ |

NP, Immunoglobulin A nephropathy (IgAN) non-progressor; P, IgAN progressor; HC, healthy controls.

^a–e^Values within a row with different superscript letters are significantly different (P < 0.05).
